# Association of the Disheveled 2 ( *DVL2* ) Gene c.2044delC Variant with Increased Risk of Canine Cleft Palate

**DOI:** 10.1002/age.70101

**Published:** 2026-04-27

**Authors:** Jonas Donner, Marjo Hytönen, Jamie Freyer, Hannes Lohi, Rebecca Chodroff Foran, Oliver P. Forman

**Affiliations:** ^1^ Wisdom Panel, Antech Diagnostics, Mars Petcare Science & Diagnostics Loveland Colorado USA; ^2^ Department of Medical and Clinical Genetics, Faculty of Medicine University of Helsinki Helsinki Finland; ^3^ Department of Veterinary Biosciences, Faculty of Veterinary Medicine University of Helsinki Helsinki Finland; ^4^ Folkhälsan Research Center Helsinki Finland

**Keywords:** canine, cleft palate, dogs, DVL2, genetics, GWAS

## Abstract

Canine congenital cleft palate is one of the most common craniofacial anomalies in dogs, characterized by a failure of the palatal shelves to fuse properly during fetal development, leading to abnormal communication between the oral and nasopharyngeal cavities. Patients with cleft palate can experience significant challenges including feeding difficulties, aspiration pneumonia, and respiratory distress, all of which can profoundly impact quality of life and, in severe cases, survival. The clinical management of cleft palate in dogs involves a combination of medical and surgical interventions. The aim of this study was to identify common genomic variants associated with cleft palate. A GWAS with 266 cases ascertained from a general population of more than 1 million dogs attending primary care veterinary clinics in the United States was used to identify an association in the region of the disheveled 2 (*DVL2*) gene variant c.2044delC, previously linked to Robinow‐like syndrome and the screw tail phenotype in Bulldogs and related breeds. The association was confirmed through genotyping of the *DVL2* variant. In summary, this study identifies the *DVL2* variant that is fixed and breed defining in several breeds as a risk factor for cleft palate in dogs.

## 1 Introduction

Canine congenital cleft palate is one of the most common craniofacial anomalies in dogs, characterized by a failure of the palatal shelves to fuse properly during fetal development, leading to an abnormal communication between the oral and nasopharyngeal cavities (Estevam et al. 2024). This condition can occur in various forms, ranging from a mild defect limited to the soft palate to a complete cleft involving both the hard and soft palates. Affected individuals can experience significant challenges, including feeding difficulties, aspiration pneumonia, and respiratory distress, which can profoundly impact their quality of life and, in severe cases, survival. The clinical management of cleft palate in dogs involves a combination of medical and surgical interventions.

The etiology of cleft palate in dogs is multifactorial, with genetic, environmental, and nutritional factors all playing potential roles. Several studies have suggested that hereditary factors contribute to the occurrence of cleft palates, particularly in brachycephalic breeds, such as the French Bull Dog, Pug, and Boston terrier (Roman et al. 2019; Estevam et al. 2022). Advances in genetic research have enabled specific genes associated with cleft palate in dogs to be identified. For example, identification of a LINE‐1 insertion in *DLX6* (OMIA ID: 001919–9615) has been associated with cleft palate and mandibular abnormalities in the Nova Scotia Duck Tolling Retriever breed (Wolf et al. 2014), and is a model for human Pierre Robin sequence. A further study in the Nova Scotia Duck Tolling Retriever revealed a frameshift mutation (c.1360_1361delAA) in the *ADAMTS20* gene (OMIA ID: 001140–9615) associated with cleft lip and palate and syndactyly, with a parallel human study identifying a suggestive association with *ADAMTS20*, highlighting its potential role in the human form of cleft lip and palate (Wolf et al. 2015).

The aim of the present study was to identify genomic variants associated with cleft palate relevant across a broad and heterogeneous cohort of single breed and mixed breed dogs, utilizing genotyping data from commercial genetic testing paired with clinical records from primary care veterinary clinics.

## 2 Materials and Methods

### 2.1 Samples

DNA samples, all originating from the United States or Mexico, were collected via commercial genetic testing of Wisdom Panel products (Antech Diagnostics, Mars Petcare Science & Diagnostics), and as a part of Optimum Wellness Plans for puppies through Banfield Pet Hospital clinics (Vancouver, WA, USA). Samples were collected through either non‐invasive buccal swabbing by dog owners or veterinary professionals, or through blood sampling by a veterinary professional. DNA extraction from whole blood and buccal swabs was performed at GeneSeek Laboratories (Neogen Co. Ltd., Lincoln, NE, USA). Consent for the use of DNA data in research was collected through the client's agreement with the terms and conditions for genetic testing with Wisdom Panel. Genetic data from Banfield‐submitted dogs were directly linked to the electronic medical records (EMRs) of the patient. Genetic data from dogs tested via consumer retail channels of Wisdom Panel were linked with the Banfield EMR by anonymized cross‐matching of pet and owner information in accordance with best practices for handling personally identifiable information (PII). The EMR was subsequently queried for dogs diagnosed with cleft palate. All types and grades of cleft palate were considered cases for the purposes of this study.

A total of 266 dogs were identified with DNA data and a diagnosis of cleft palate at any time since birth. A total of 506 controls were defined as dogs without a recorded diagnosis of cleft palate in the Banfield EMR. Breed assignment was based on a reference panel of over 27 000 dogs of known ancestry from more than 50 countries and ascertained using the BCSYS Local Ancestry Classifier algorithm (Garrigan et al. 2023). For the purposes of this study, mixed breed dogs were defined as dogs with no greater than 75% of their ancestry from a single breed (equivalent to a maximum of 3 grandparents of the same breed).

### 2.2 Microarray Genotyping

Genotyping was performed at GeneSeek Laboratories following manufacturer‐suggested standard protocols on a custom 100 k Illumina Infinium XT microarray (Illumina Inc., San Diego, CA, USA). The microarray contained tests for known canine Mendelian diseases and traits, as well as genome‐wide coverage, and had been designed and validated for use as previously described (Donner et al. 2023). Samples achieving less than a 97% genotyping call rate across the full marker set were excluded from the study.

### 2.3 Statistical Analyses

A total of 95 165 array variants were available for analysis prior to filtering. After filtering and removal of samples with greater than 5% missing data, SNPs with a minor allele frequency ≤ 1%, variants with greater than 5% missing data, and SNPs with an absolute difference in call rate between males and females > 2.5%, a total set of 90 788 variants remained for the all‐breed and mixed breed GWAS and 90 104 for the mixed breed GWAS. Genome‐wide association testing was performed using a linear mixed‐model approach with the software package GEMMA v0.98.5 (Zhou and Stephens 2012), including a centered relatedness matrix. Manhattan and QQ plots were created using the R package qqman v0.1.9 (Turner 2018). The analyses were carried out within the Databricks cloud‐based data analytics system. The significance of the *DVL2* gene variant frequency distribution between groups of dogs was evaluated using a standard allelic association analysis using PLINK (Purcell et al. 2007). All genome coordinates refer to the CanFam4 genome build (UU_Cfam_GSD_1.0, GCF_011100685.1).

### 2.4 Whole‐Genome Sequencing

Whole‐genome sequencing was performed using DNA from the buccal swabs collected through commercially available DNA testing. Whole‐genome sequencing was performed using a standard methodology to achieve a target read depth of 30× on an Illumina NovaSeq at Neogen Inc., Lincoln, Nebraska, United States. The data were analyzed using the Illumina Dragen pipeline and aligned to the CanFam4 genome assembly. Variants were annotated using SNPeff (Cingolani, Platts, et al. 2012), and statistical analysis was performed using SNPsift (Cingolani, Patel, et al. 2012). Samtools 1.13 (Danecek et al. 2021) was used to filter the variant set to include those within the specific chromosomal region of interest.

### 2.5 Follow‐Up Genotyping

The *DVL2* variant was genotyped with standard PCR and Sanger sequencing using the primers designed by Mansour et al. 2018 (Mansour et al. 2018): 5′‐CGGCTAGCTGTCAGTTCTGG‐3′ (forward) and 5′‐CAGTGAGTCTGAGCCCTCCA‐3′ (reverse). Amplified products were treated with exonuclease I (New England Biolabs) and rapid alkaline phosphatase (Roche Diagnostics) and sequenced by capillary electrophoresis (Applied Biosystems ABI3730XL DNA Analyzer) at the Institute for Molecular Medicine Finland FIMM core facility of University of Helsinki. Sequences were analyzed using UGENE v52.0 software (Okonechnikov et al. 2012).

## 3 Results

### 3.1 Genome‐Wide Association Study

**FIGURE 1:**
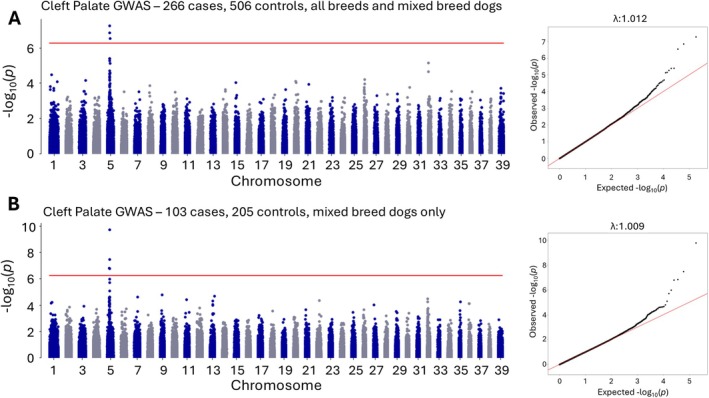
GWAS analyses of (A) 266 cases and 506 controls (all single breed and mixed breed dogs) and (B) 103 cases and 205 controls (mixed breed dogs only). A Bonferroni corrected significance line is shown in red (0.05/number of SNPs analyzed). QQ plots are shown to the right of Manhattan plots demonstrating control for population stratification.

An initial GWAS was performed using all dogs diagnosed with cleft palate and approximately 2× randomly selected age‐matched controls. A genome‐wide significant signal was observed on chromosome 5 with the most associated SNP at chr5:33309585 (3.25 × 10^−8^) shown in Figure 1. Although a Lambda value of 1.012 was observed, indicating reasonable adjustment for population stratification, cases were heavily skewed toward the French Bulldog breed. A cleft palate GWAS including only the French Bulldog breed yielded no genome‐wide significant results, suggesting that the disease‐associated variant may be fixed in the breed population (data not shown). A mixed breed GWAS was performed including dogs with less than 75% of any one breed in their overall ancestry, equivalent to one grandparent from a different breed origin. Despite the smaller case set, a signal of increased significance was observed at the same chromosome 5 locus with the most associated SNP at chr5:33082766 (1.75 × 10^−10^), Lambda 1.009 (Figure 1). The leading positional candidate gene with the closest proximity to the most associated GWAS SNP was *DVL2*, with variants in this gene previously associated with a screw tail phenotype in dogs and Robinow syndrome in humans (Mansour et al. 2018; Patton and Afzal 2002). The breed composition of dogs in each GWAS can be found in File S1.

### 3.2 Whole‐Genome Sequencing

Three clinical cases of cleft palate were selected for whole‐genome sequencing (one English Bulldog, one American Staffordshire Terrier and one Boston Terrier), all of which were homozygous for the associated alleles of most significant SNPs of the two GWAS. Cases with different breed backgrounds were chosen to avoid bias toward a particular breed and to include at least one breed that was not fixed for the *DVL2* c.2044delC variant. The three case genomes were compared with a general group of 80 controls that were clear of a cleft palate diagnosis, and whole‐genome‐sequenced for other unrelated projects. Given a genetic risk factor was being investigated, rather than a fully penetrant autosomal recessive condition where region boundaries can be defined by recombinational breakpoints, an empirical 4 Mb region around the most significant SNP was defined for analysis purposes. Variants were ranked based on *p*‐value (SNPsift) and filtered according to consequence predictions (SNPeff). A total of 29 345 variants were identified. The most significant high impact variant was *DVL2* c.2044delC. Three highly associated moderate impact missense variants were also identified; however, subsequent SIFT (Vaser et al. 2016) analysis indicated these variants to be likely tolerated and were therefore excluded from further analysis. A full list of genome sequenced dogs, variants and SIFT analysis of excluded variants can be found in File S2.

### 3.3 Follow‐Up Genotyping of the *DVL2* Variant

As the previously identified *DVL2* variant was not directly included in the microarray used for the present study, we pursued separate genotyping to assess its association with cleft palate. We genotyped the variant in 47 mixed breed dogs (< 75% of any one breed) from the original GWAS case cohort for which DNA was still available, and in 113 additional randomly selected mixed breed control dogs. Association analysis of the *DVL2* variant along with the most significant SNPs observed across both GWAS analyses resulted in the *DVL2* variant achieving the lowest probability value. It was therefore determined as the strongest candidate variant for causing an increased risk of cleft palate formation (Table 1).

**TABLE 1:** Analyses of leading SNPs in the *DVL2* region showing results from the GWAS (GWAS all and GWAS mixed) and PLINK association analysis for a subset of the samples genotyped for the *DVL2* variant and leading SNP variants. Most associated SNPs are highlighted.

SNP ID	chr:position	GWAS ALL	GWAS MIXED	PLINK MIXED
BICF2G630182200	5:30609834	5.37E‐06	2.54E‐05	1.43E‐11
BICF2G630182371	5:31046888	4.13E‐06	5.97E‐05	2.11E‐08
BICF2G630182460	5:31201621	3.06E‐05	1.48E‐07	1.98E‐12
BICF2G630182626	5:31767630	5.05E‐05	8.11E‐06	3.50E‐11
***DVL2* screwtail variant**	5:32401931	—	—	**9.03E‐21**
**BICF2G630183073**	5:33082766	1.43E‐07	**1.75E‐10**	3.39E‐10
**BICF2P731728**	5:33309585	**5.45E‐08**	1.98E‐06	9.52E‐13
BICF2G630183931	5:34706239	2.87E‐07	3.43E‐08	4.01E‐14
BICF2G630183965	5:34925128	7.51E‐06	1.63E‐07	1.72E‐11
**BICF2G630184103**	5:35242543	4.46E‐04	1.08E‐06	2.92E‐12

To demonstrate the association in a breed where *DVL2* c.2044delC associated traits are not a defining feature, genotype data from American Staffordshire Terriers and American Staffordshire terrier mixes were assessed separately. 19 out of 27 cases in this subset were homozygous for *DVL2* c.2044delC, compared with zero out of 15 controls (Table 2). A full list of dogs with breed compositions can be found in File S1.

**TABLE 2:** *DVL2* c.2044delC genotype data for American Staffordshire terrier cases and controls. None of the control samples were homozygous for the c.2044delC variant, in comparison to 19 of the 27 cases. Fisher exact test; *p* = 7.3 × 10^−7^.

	wt/wt	wt/dvl2	dvl2/dvl2	Total
Cases	2	6	19	27
Controls	11	4	0	15

## 4 Discussion

This study formally establishes an association between cleft palate and a previously identified frameshift variant in the *DVL2* gene, that has been linked to brachycephaly and caudal vertebral anomalies (Mansour et al. 2018). As brachycephalic breeds are overrepresented among dogs with orofacial clefts, and many of these are breeds fixed for *DVL2* c.2044delC, the use of a diverse study cohort of mixed breed and single breed dogs was essential in helping to identify this association. Concerns related to the effects of population stratification in GWAS studies with dogs are common, due to their unique population structure. However, this study offers some reassurance that software packages such as Gemma provide excellent control for population stratification as demonstrated by a single peak of significance being identified, neither of which were the *BMP3* or *SMOC2* loci associated with brachycephalic breeds, despite their overrepresentation in the total case set (Schoenebeck et al. 2012; Marchant et al. 2017). Analysis of American Staffordshire terrier and American Staffordshire terrier mixes only provided further evidence of the association between *DVL2* c.2044delC and cleft palate, in a less stratified subset.

Variants in *DVL2* cause Robinow syndrome in humans, a rare genetic disorder characterized by a range of distinctive features, including facial dysmorphism (broad, flattened forehead, wide‐set eyes, and a small, underdeveloped chin), skeletal abnormalities, and, in some cases, cleft palate (Conlon et al. 2021). *DVL2* variants have also been associated with non‐syndromic human cleft palate (Mostowska et al. 2012). This makes *DVL2* c.2044delC a logical candidate for causing increased risk of cleft palate in dogs and adds to the list of morphological variants with pleiotropic effects, such as the *HMGA2* size associated variant and the *FGF4* retrogene insertion, among others (Brown et al. 2017; Blades et al. 2022; Rimbault et al. 2013).

In conclusion, the association of the *DVL2* c.2044delC frameshift variant with cleft palate is well supported and adds to the previously associated phenotypes in dogs such as wide‐set eyes and cork‐screw tail. This confirmatory discovery establishes breeds that are fixed for the screw‐tail phenotype as inherently at risk of cleft palate. Although an increased incidence of cleft palate in brachycephalic breeds is well established (Roman et al. 2019), this finding suggests that the screw‐tail/ *DVL2* c.2044delC variant, which is common in dome head‐shaped breeds, is likely driving the risk of cleft palate.

## Author Contributions

**Jonas Donner:** conceptualization, methodology, investigation, formal analysis, writing – original draft, writing – review and editing. **Rebecca Chodroff Foran:** writing – review and editing, project administration, supervision. **Jamie Freyer:** methodology, investigation, data curation, writing – review and editing. **Oliver P. Forman:** conceptualization, methodology, data curation, investigation, validation, formal analysis, supervision, project administration, writing – review and editing, writing – original draft. **Marjo Hytönen** **:** investigation, formal analysis, writing – original draft, writing – review and editing. **Hannes Lohi:** supervision, funding acquisition, writing – review and editing.

## Funding

This study was partially supported by the Jane and Aatos Erkko Foundation.

## Ethics Statement

Specific animal research ethics approval was not required as all analyses were carried out on DNA extracted from owner‐collected, non‐invasive cheek swab samples or from blood/cheek swab samples collected at certified veterinary clinics in accordance with international standards for animal care. All dog owners provided consent for the use of their dogs' DNA samples for research purposes.

## Conflicts of Interest

J.D., J.F., R.C.F., and O.P.F. are employees of Wisdom Panel (Antech Diagnostics, Mars Petcare Science & Diagnostics) which offers canine genetic testing services.

## Supporting information


**File S1:** Full genetically determined breed ancestry of the cases and controls used in the GWAS.

**File S2:** Variants identified through whole‐genome sequence analysis, including SIFT scores of segregating moderate impact SNPs, and a summary of genome sequenced dogs.

## Data Availability

The data that supports the findings of this study are available in the Supporting Information of this article.
